# Unsupervised deep learning framework for temperature-compensated damage assessment using ultrasonic guided waves on edge device

**DOI:** 10.1038/s41598-024-54418-w

**Published:** 2024-02-14

**Authors:** Pankhi Kashyap, Kajal Shivgan, Sheetal Patil, B. Ramana Raja, Sagar Mahajan, Sauvik Banerjee, Siddharth Tallur

**Affiliations:** 1 Department of Electrical Engineering (EE), IIT Bombay, Mumbai, 400076 India; 2 Department of Civil Engineering (CE), IIT Bombay, Mumbai, 400076 India

**Keywords:** Electrical and electronic engineering, Techniques and instrumentation

## Abstract

Fueled by the rapid development of machine learning (ML) and greater access to cloud computing and graphics processing units, various deep learning based models have been proposed for improving performance of ultrasonic guided wave structural health monitoring (GW-SHM) systems, especially to counter complexity and heterogeneity in data due to varying environmental factors (e.g., temperature) and types of damages. Such models typically comprise of millions of trainable parameters, and therefore add to cost of deployment due to requirements of cloud connectivity and processing, thus limiting the scale of deployment of GW-SHM. In this work, we propose an alternative solution that leverages TinyML framework for development of light-weight ML models that could be directly deployed on embedded edge devices. The utility of our solution is illustrated by presenting an unsupervised learning framework for damage detection in honeycomb composite sandwich structure with disbond and delamination type of damages, validated using data generated by finite element simulations and experiments performed at various temperatures in the range 0–90 °C. We demonstrate a fully-integrated solution using a Xilinx Artix-7 FPGA for data acquisition and control, and edge-inference of damage. Despite the limited number of features, the lightweight model shows reasonably high accuracy, thereby enabling detection of small size defects with improved sensitivity on an edge device for online GW-SHM.

## Introduction

Ultrasonic guided waves (GW) have the ability to propagate long distances without significant attenuation. GW based structural health monitoring (SHM) systems are highly sensitive to damages, and require minimal instrumentation of sensors on the structure to be monitored. Over the years, many studies has been reported on such systems, and a vast body of literature is available on GW SHM^1^. However, unlike vibration condition based monitoring of rotating machinery^2^, the industrial acceptance towards replacement of periodic nondestructive testing (NDT) with such permanently deployed SHM systems has been quite limited^3^. The primary hurdle in this regard is the impact of variations in environmental and operating conditions (EOCs) on GW propagation. The amplitude and group velocity of different GW modes have distinct temperature-dependent coefficients, and therefore assessing damages in presence of time-varying EOCs imposes a significant challenge^4^. Recently, data-driven damage assessment methods using machine learning (ML) techniques have been proposed to overcome this limitation, by identifying patterns hidden in the features computed from GW recordings^5^. Such reports include methods based on artificial neural networks (ANNs) and support vector machines (SVMs)^6^, principal component analysis (PCA)^7^, singular value decomposition (SVD)^8,9^, Gaussian mixture models (GMMs)^10,11^ etc. On the other hand, deep learning techniques for GW based SHM eliminate the dependencies on domain knowledge for computation of features, by utilising the deep learning models themselves for feature computation and classification^12–14^. Recently, Sawant et al.^15^ demonstrated a method which utilizes supervised convolutional neural networks (CNNs) to learn the features of GW signals acquired from healthy and damage structures for varying temperature conditions, combined with GMMs for damage index computation and localisation. Unlike such supervised methods that require labeled data collected for healthy as well as damaged structures, unsupervised learning approaches offer greater utility, as most practical applications of SHM systems will result in generation of significantly higher amount of data for healthy operating conditions as compared to damaged structure. Recently reported unsupervised learning demonstrations for GW-SHM include convolutional denoising autoencoder (AE) for temperature compensation^16^ (albeit, without damage assessment) and deep AEs operating on time-domain GW data obtained on composite panels^17,18^. Typical deep learning architectures containing convolutional layers result in millions of trainable parameters and require large amount of computational resources and take long time for training. Cloud based data storage and inference may be the most suitable option for meeting these requirements; however, the added cost of data transmission and storage, latency, and security and privacy aspects potentially limit their applications. Hence, there is a need for development of data driven unsupervised algorithms for damage assessment, that could be implemented in edge devices such as microcontrollers and field programmable gate arrays (FPGAs) used for data acquisition.

TinyML has emerged as a promising solution for seamless development of light-weight ML models on a variety of microcontrollers, resulting in a vast body of work on memory-efficient and low-power edge-implementation of ML applications^19,20^. The ML models are typically trained on a workstation, and deployed on edge devices using device-specific libraries and open-source frameworks such as TensorFlow Lite Micro (TFLM)^21,22^. While this enables deployment of models on low-cost microcontrollers with limited resources (e.g. single-board platforms such as Arduino), ML models typically require to be retrained in the field to accommodate drift in the sensor recordings, and therefore more powerful edge devices such as Raspberry Pi single board computer or FPGA platforms may be more suitable for practical applications^23,24^. FPGAs offer design flexibility in GW-SHM applications, by allowing seamless reconfiguration of the ML model architecture and hardware implementation and acceleration of the multiply-accumulate (MAC) operations involved in data processing, without hardware redesign. The high speed clock offered (tens of MHz and higher) by most FPGAs also makes them suitable for interfacing with high speed digital to analog converters (DACs) and analog to digital converters (ADCs) for the GW transmitters and receivers,respectively. Aranguren et al. recently demonstrated an FPGA-based GW-SHM system for detection of progressive damage in aircraft structures^25^. Malviya et al. demonstrated an FPGA-based implementation of light-weight convolutional AE model for anomaly detection in vibration-based monitoring, validated using the Airbus SAS helicopter accelerometer dataset^24^. Thus, while embedded systems with edge-learning have been developed for a variety of applications, there have been no such reports for GW SHM to counter EOC variations. Moreover, models that leverage convolutional layers to learn features from vast amounts of training data are impractical due to low memory availability on such embedded systems.

In this paper, we demonstrate a TinyML-enabled unsupervised learning framework for GW-SHM, implemented on the Xilinx Artix-7 FPGA, to realize a fully-integrated SHM system for data acquisition and damage assessment (identification and localisation). From a network of 8 PZT sensors covering an undamaged and two damaged portions of a panel made of honeycomb composite sandwich structure (HCSS), GW data are recorded at different temperatures from 0 to ${90}\,^{\circ }\hbox {C}$. A light-weight neural network is trained on features obtained from data collected on an undamaged panel, and is then tested on independently collected data from undamaged as well as damaged portions of the panel. The features comprise of various parameters obtained from time-domain GW signal recordings. The light-weight model is deployed using TensorFlow Lite framework on the MicroBlaze$^{\circledR }$ reduced instruction set computer (RISC) processor core in the Xilinx Artix$^{\circledR }$-7 FPGA for damage assessment at the edge. The efficacy of this method is demonstrated using GW data from a network of PZT sensors on an HCSS panel with teflon release film (TRF) and lack of film adhesive (LFA) damages, data collected by experimental measures at various temperatures (from 0 to ${90}\,^{\circ }\hbox {C}$). A parametric study is also done using data generated by finite element (FE) simulations to see the model performance for detection of damages with different sizes. The mean square error (MSE) of the reconstructed signal is used as signal difference coefficient (SDC) for damage identification. This manuscript presents extended results based on preliminary work presented at 2023 IEEE EFTF/IFCS^26^. The system presented in this work is a promising development towards fully-integrated data-driven GW-SHM solution. The key contributions of this work are:

- *TinyML based unsupervised learning model deployed on edge device for GW-SHM*: We present a light-weight unsupervised learning model $(<10,000$ trainable parameters) consisting of only densely connected layers, for processing GW ultrasonic time-series signals represented by a set of sixteen features. The small size of the model makes it possible to deploy the model for inference at the edge on a low-cost FPGA (Xilinx Artix-7)
- *Temperature-compensated damage assessment in honeycomb composite sandwich structure*: The light-weight model was trained and tested on experimental data collected on a HCSS panel in presence of large temperature variation (0–${90}\,^{\circ }\hbox {C}$), and is able to distinguish changes in signal due to damage from changes due to temperature variation with accuracy in excess of 95 %
- *Integrated solution using low-cost FPGA for data acquisition and damage inference*: The custom-developed SHM system presented in this work uses a Xilinx Artix-7 FPGA for PZT transduction, data acquisition and recording, model inference and damage assessment, with power consumption of 0.336 W and inference time of approximately ${900}\,\upmu \hbox {s}$.

## Methodology

### Overview of proposed method

**Figure 1:**
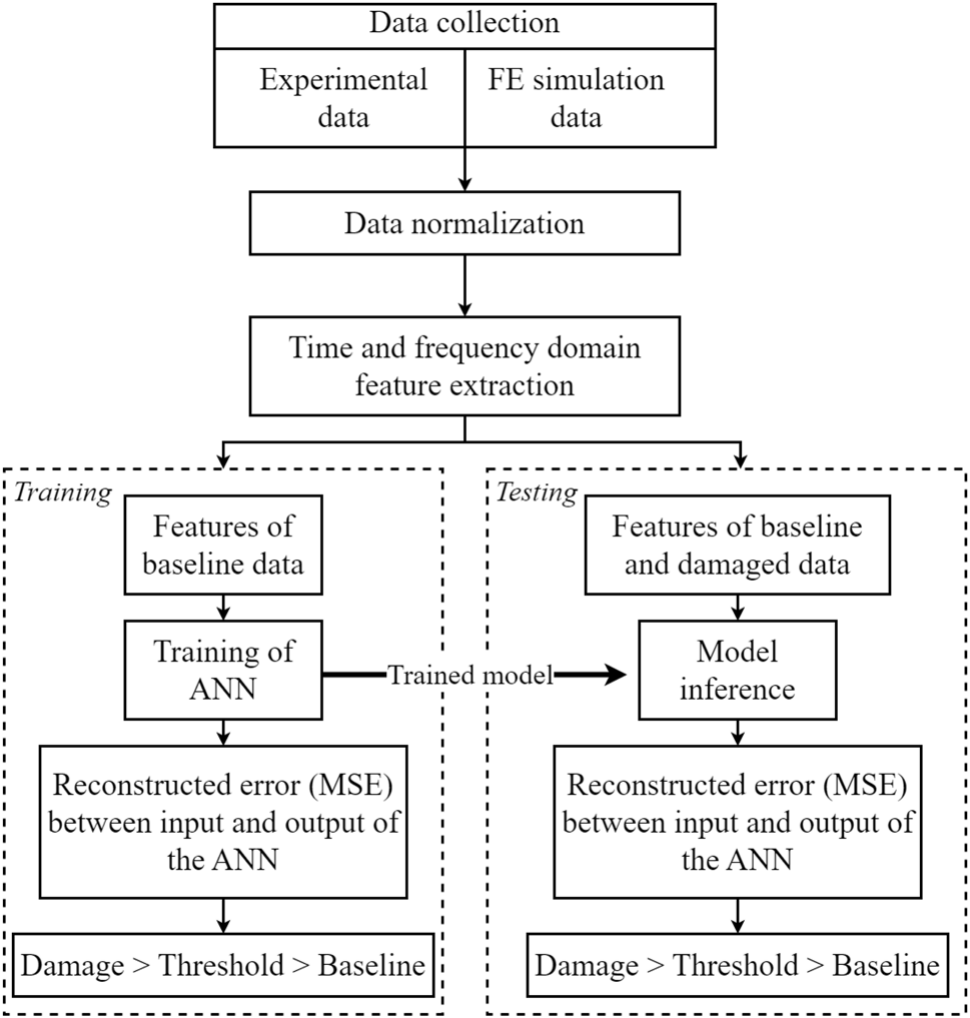
Overview of the proposed methodology.

The proposed damage assessment method broadly comprises of three steps: (i) feature extraction, (ii) training of unsupervised learning model, and (iii) edge implementation of trained model. Figure 1 shows the overview of our proposed methodology. First, time-series data were collected from a structure instrumented with PZT sensors, for healthy (undamaged) and damaged conditions. Data were generated using FE simulations and experimental setup for temperatures varying from 0 to ${90}\,^{\circ }\hbox {C}$. Next, features are computed from the signals, based on the sensitivity to damage, computational cost and memory requirement associated with computing the features for edge implementation. Note that the features can be computed from time and frequency domain representations of the signals. In this work, we have only focused on the use of time-domain features, as they are simpler to compute and do not introduce any significant computation overhead on the embedded system. To study the performance of the model in presence of large errors, white noise and pink noise were added to the data to obtain signal to noise ratio (SNR) of 20 dB. Next, an artificial neural network (ANN) was trained on the manually computed set of features, obtained using data collected for undamaged (baseline) structure under various temperature conditions. When the trained model is tested on data obtained from damaged panels, the paths containing damage located between the transmitter and receiver positions produce higher reconstruction error (mean squared error, MSE) for the model prediction, which in turn is used to identify damage. The trained model is deployed on a Xilinx Artix-7 FPGA for damage inference using TensorFlow Lite framework operating in the MicroBlaze RISC processor in the FPGA. The FPGA is also used for data acquisition and feature computation, and thus serves as an end-to-end smart GW-SHM solution.

### Experimental data acquisition

In this work, we have evaluated the proposed unsupervised learning method for damage assessment of a HCSS panel, which is an advanced composite structure widely used in aerospace, automotive and marine industries. The panel of lateral dimensions $1\,\hbox {m} \times 1.2\,\hbox {m}$ was manufactured by sandwiching two composite face sheets with a 12.7 mm thick aluminum honeycomb core between the face sheets. The face sheets comprised of six 0.125 mm thick unidirectional layers with a layer-wise orientation of $[0, +60, -60, -60, +60, 0]$ and were separately cured and bonded to the core. Disbond was artificially created between the core and face sheet by removing the film adhesive in a region of dimensions $30\,\hbox {mm} \times 30\,\hbox {mm}$, and delamination was created between the second and third layer of the face sheet adjacent to the core by inserting a thin layer of teflon release film of dimensions $30\,\hbox {mm} \times 30\,\hbox {mm}$. The effect of temperature variation on the characteristics of GW signals for disbond (LFA) and delamination (TRF) damages in this structure was extensively characterised and reported in our previous work^27^. The amplitude and group velocity of the fundamental anti-symmetric (A0) mode were found to increase in presence of disbond and decrease in presence of face sheet delamination. However, the amplitude of A0 mode reduced linearly with increasing temperature for both healthy and damaged cases. The variation in amplitude of the A0 mode due to temperature makes it difficult to use this property to evaluate the presence of damage, especially in presence of large noise.

**Figure 2:**
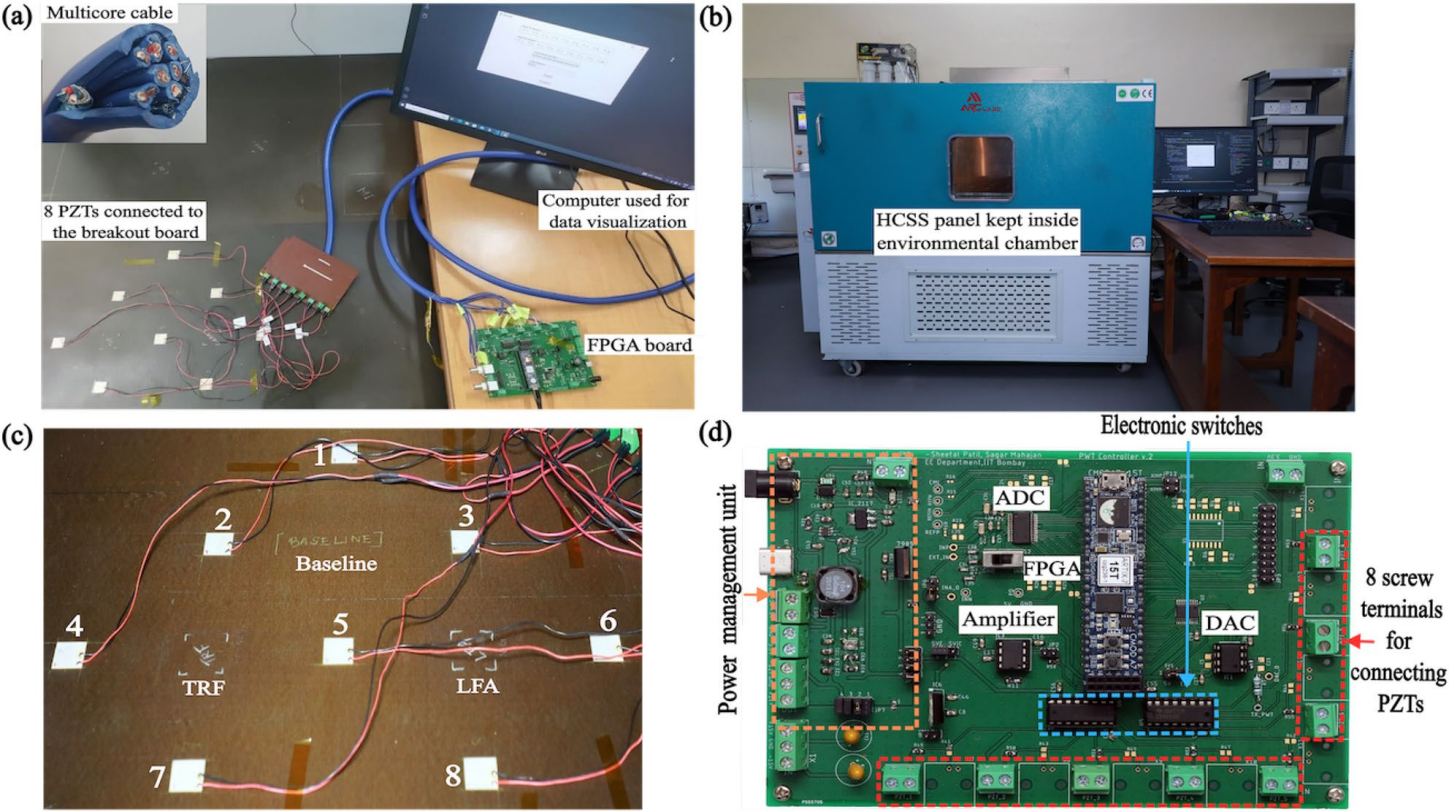
Overview of the experimental setup. (**a**) PZT network on HCSS structure connected to FPGA board using a shielded multicore cable. Inset: 8 pairs of shielded cables in the multicore cable. (**b**) Environmental chamber used for temperature control. (**c**) HCSS panel showing positions of PZT transducers and damages (TRF and LFA) and undamaged (baseline) locations. (**d**) FPGA enabled embedded system for data acquisition and damage assessment.

Ultrasonic GW data from the structure were recorded by attaching a network of eight PZT-5H transducers on one of the face sheets of the structure (Fig. 2a). Each transducer is of dimension $30\,\hbox {mm} \times 30\,\hbox {mm} \times 0.4\,\hbox {mm}$. The transducers were placed in such a manner so that the distance between transmitter and receiver remains 180 mm for any horizontal or vertical path in a unit cell. A multicore cable of length 3 m with electromagnetic shielding was used to connect the PZTs with the FPGA board. Electromagnetic shielding prevents, or at the very least, reduces the coupling of undesired radiated electromagnetic energy in systems and cables. This is necessary in our experiment because the FPGA board is kept outside an environmental chamber (Arcade Scientific Instruments Pvt. Ltd., Fig. 2b) used to record ultrasonic GW signals on the HCSS panel, and thus requires analog voltage signals from the PZTs to be connected with a long cable, that is susceptible to electromagnetic interference. A closer view of the region of interest on the HCSS panel (Fig. 2c) shows that the TRF damaged portion lies at the center of unit cell with PZTs numbered 2, 7 and 4, 5, and LFA damaged portion lies at the center of unit cell with PZTs numbered 3, 8 and 5, 6. Similarly, the region at the intersection of horizontal and vertical paths in unit cell with PZTs numbered 1, 5 and 2, 3 does not have any damage and is considered as baseline region. We collected time-series data from the above mentioned vertical and horizontal paths. Ultrasonic GW data were recorded for these horizontal and vertical paths by placing the HCSS panel in the environmental chamber and varying temperature from 0 to ${90}\,^{\circ }\hbox {C}$, in intervals of ${5}\,^{\circ }\hbox {C}$.

Figure 2d shows the portable embedded system built for GW ultrasonic signal transduction, data acquisition, and processing. The system is based on a low-cost FPGA (Xilinx Artix-7 XC7A15T-1CPG236C, Digilent Cmod A7-15 T module). The FPGA is interfaced with a 12 bit, parallel input, multiplying digital to analog converter (DAC) - Texas Instruments DAC7821, with a transimpedance amplifier (Texas Instruments TL072) to generate the $5-$cycle Hanning pulse actuation voltage centered at 75 kHz with peak-to-peak amplitude of 10 V. The receiver PZT output is amplified using Texas Instruments INA128 instrumentation amplifier and digitised using Maxim Integrated MAX-1426 analog to digital converter (ADC) with 10 bit resolution and 10 Msps sampling rate. The printed circuit board (PCB) also consists of an on-board power supply module with voltage regulators for generating necessary supply rails for the amplifier and data converter ICs, so that the entire board can be powered with a USB type-C charger. Voltage controlled switches implemented with Texas Instruments CD4051 allow digital selection of the PZTs to be connected as transmitter and receiver to the signal chain. The system is capable of storing 4096 samples obtained at 10 Msps i.e., ${409.6}\,\upmu \hbox {s}$ for each channel in the memory (Block RAM) in the FPGA. A graphical user interface (GUI) created using Python is used for system control and configuration, and operating the experiment (selection of PZT channels, visualising GW data and saving data to file). To incorporate uncertainties caused by variation in other environmental and operating conditions that one may expect in the field, we also added white noise and pink noise to the data to obtain SNR of 20 dB^15^ (note that the SNR of the data recorded with the system described above is >50 dB). Fifty noise-augmented copies were generated from each data recording through this exercise.

**Figure 3:**
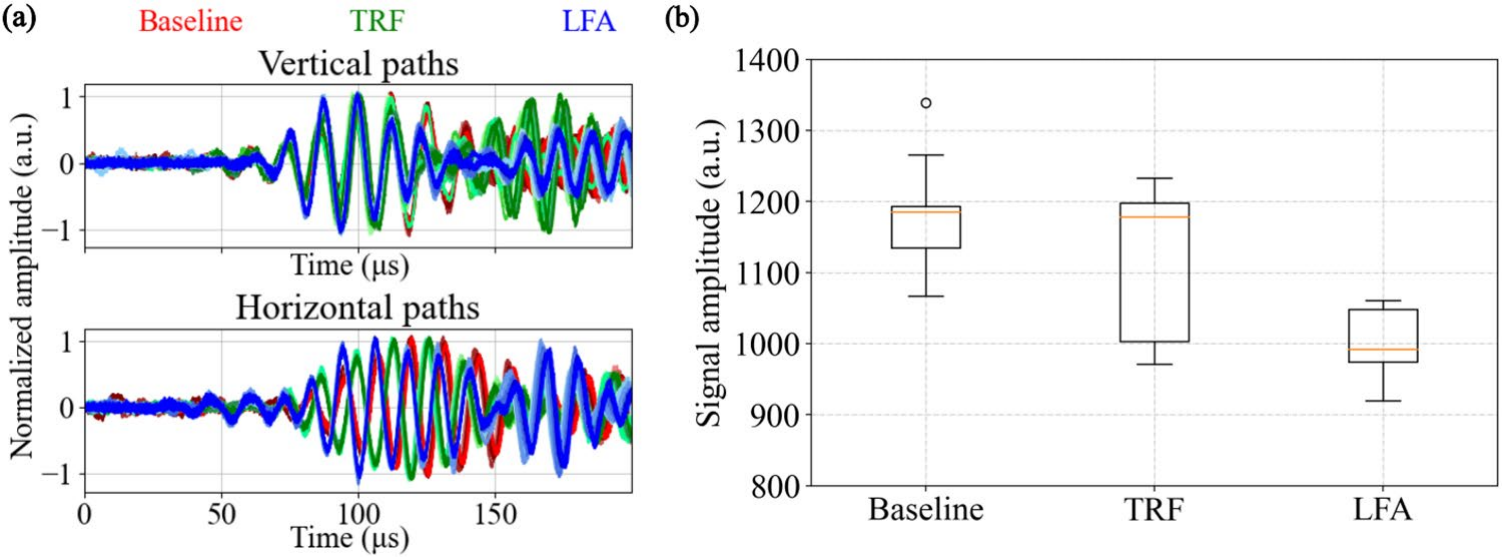
(**a**) Noise-augmented data series recorded from 0 to ${90}\,^{\circ }\hbox {C}$, for all horizontally and vertically oriented paths passing through baseline, TRF and LFA regions. Lighter shades of colour represents lower temperature and darker shades represent progressively higher temperatures. (**b**) Variation in amplitude of A0 mode due to temperature, shown as distribution for baseline, TRF and LFA damages.

Figure 3a shows a plot of representative noise-augmented data gathered at all temperatures in the range of 0–${90}\,^{\circ }\hbox {C}$ for vertical and horizontal paths in all unit cells up to ${200}\,\upmu \hbox {s}$. The traces labelled as *Vertical paths* show signal data collected along paths connecting PZTs oriented vertically: i.e. between PZT 1 and PZT 5 for baseline region, between PZT 2 and PZT 7 for TRF, and between PZT 3 and PZT 8 for LFA. Similarly, the *Horizontal paths* traces show signal data collected along paths connecting PZTs oriented horizontally: i.e. between PZT 2 and PZT 3 for baseline region, between PZT 4 and PZT 5 for TRF, and between PZT 5 and PZT 6 for LFA. Data acquired at higher temperatures are shown using deeper colour shades, whereas data at lower temperatures are indicated by lighter colour shades. Figure 3b provides insights into variation of amplitude of the A0 mode with temperature for noise-augmented data obtained from each region. This plot combines data from both vertical and horizontal paths within each region. It is not possible to distinguish the baseline and TRF signals merely from inspection of signal amplitude in presence of temperature variation and added noise. Apart from amplitude variation, we also note that the phase of the signal for the A0 mode (indicative of group velocity) also changes response to temperature fluctuations. The combined influence of temperature-induced amplitude and phase variations complicates the straightforward interpretation of damage solely based on these parameters.

### Data generation through finite element method (FEM) simulations

The experimental sample used in this study contained only a few damages, one each of TRF and LFA damage. Therefore, to study the sensitivity of the method proposed in this work for damage assessment for various sizes of defects, additional data were generated through FEM simulations. Building a 3D model of the entire panel (lateral dimensions $1\,\hbox {m} \times 1.2\,\hbox {m}$) is computationally expensive and therefore impractical. Since the GW propagation paths used in our study are straight lines, we employed a 2D model to simplify this exercise. GW propagation in a $1200\,\hbox {mm} \times 14.2\,\hbox {mm}$ HCSS with surface mounted PZTs of dimensions $20\,\hbox {mm} \times 0.7\,\hbox {mm}$ was analysed using FE simulations (COMSOL Multiphysics 5.5).

The simulation model was set up in a manner similar to the method described in our earlier work^27^. A brief overview of the method is presented here. Three cases were considered: (a) no damage, (b) disbond (LFA), and (c) TRF (delamination). The disbond was created by removing adhesive layer between the skin and the core, and delamination was created by inserting a Teflon release film layer with a thickness of 0.01 mm between second and third layers of the face sheet. The damages were located in the centre of the transmitter-receiver path. Data were generated for various sizes of damage: ${5}\,\hbox {mm}$, ${10}\,\hbox {mm}$, ${15}\,\hbox {mm}$, and ${20}\,\hbox {mm}$ and at various temperatures in the range of 30–${90}\,^{\circ }\hbox {C}$ in intervals of ${10}\,^{\circ }\hbox {C}$. A stationary study was first carried out where the temperature of the HCSS panel was gradually increased from initial room temperature to the desired set-point using *Thermal Expansion* sub-node in material properties, and temperature-dependent material properties for the HCSS were derived. If the temperature is suddenly increased in a single step, the results of the stationary study will not converge. To avoid this, the temperature is gradually changed from room temperature to desired temperature^27^. Temperature-dependent material properties are derived layer-wise using the Chamis relation^28^, and change in material properties of PZT due to temperature were calculated using equations developed by Roy et al.^29^

Next, time-dependent study was carried out to simulate GW propagation in HCSS at elevated temperature, using *Structural Mechanics* module coupled with *Piezoelectric* interaction physics analysed using time-implicit study. Mapped quadrilateral mesh elements were used in the FE model, with maximum and minimum element sizes of 1 mm and 0.01 mm, respectively. At 75 kHz actuation frequency, the phase velocity of the A0 mode (the mode employed in this work) is 1.061 km/s, and the wavelength is calculated to be 14.44 mm. Accurate simulation of Lamb wave propagation in implicit analysis requires at least ten mesh elements per wavelength, and this criterion is thus satisfied. Low reflecting boundary condition was specified at the outer edges of HCSS to reduce reflections from edges, and Rayleigh damping was added for the composite face sheet and the core to account for damping of the GWs during propagation. The material properties used for HCSS are provided in Table 1 and elastic properties of the PZT are provided below^27^:

$$\begin{aligned}{} & {} {[}\varepsilon ] = \begin{bmatrix} 29.44 &{} 0 &{} 0 \\ 0 &{} 29.44 &{} 0 \\ 0 &{} 0 &{} 7.48 \\ \end{bmatrix} \times 10^{-9} \ \text {F/m},\\{} & {} {[}C]=\begin{bmatrix} 127.21 &{} 80.21 &{} 84.67 &{} 0 &{} 0 &{} 0 \\ 80.21 &{} 127.21 &{} 84.67 &{} 0 &{} 0 &{} 0 \\ 84.67 &{} 84.67 &{} 117.41 &{} 0 &{} 0 &{} 0 \\ 0 &{} 0 &{} 0 &{} 22.99 &{} 0 &{} 0 \\ 0 &{} 0 &{} 0 &{} 0 &{} 22.99 &{} 0 \\ 0 &{} 0 &{} 0 &{} 0 &{} 0 &{} 23.47 \end{bmatrix} \times 10^9 \ \text {N/m}^2,\\{} & {} \hbox {and}\\{} & {} {[}e]=\begin{bmatrix} 0 &{} 0 &{} 0 &{} 0 &{} 17.04 &{} 0 \\ 0 &{} 0 &{} 0 &{} 17.04 &{} 0 &{} 0 \\ -6.63 &{} -6.63 &{} 23.24 &{} 0 &{} 0 &{} 0 \end{bmatrix} \times 10^{-3} \ \text {N/m/V} \end{aligned}$$

**Table 1:** Material properties of HCSS.

Material	$E_1$ (GPa)	$E_2$ (GPa)	$E_3$ (GPa)	$G_{12}$ (GPa)	$G_{23}$ (GPa)	$G_{13}$ (GPa)	$\nu _{12}$	$\nu _{13}$	$\nu _{23}$	$\rho$ (kg/m$^3$)	t (mm)
Lamina (face sheet)	135	9	9	4.5	5.5	4.5	0.29	0.45	0.29	1780	0.125
Core	0.0802	0.0802	1.6121	0.0321	0.0964	0.0964	0.25	0.025	0.025	32	12.7
Adhesive	0.0487	0.0487	0.0487	0.0174	0.0174	0.0174	0.40	0.40	0.40	1250	0.01

**Figure 4:**
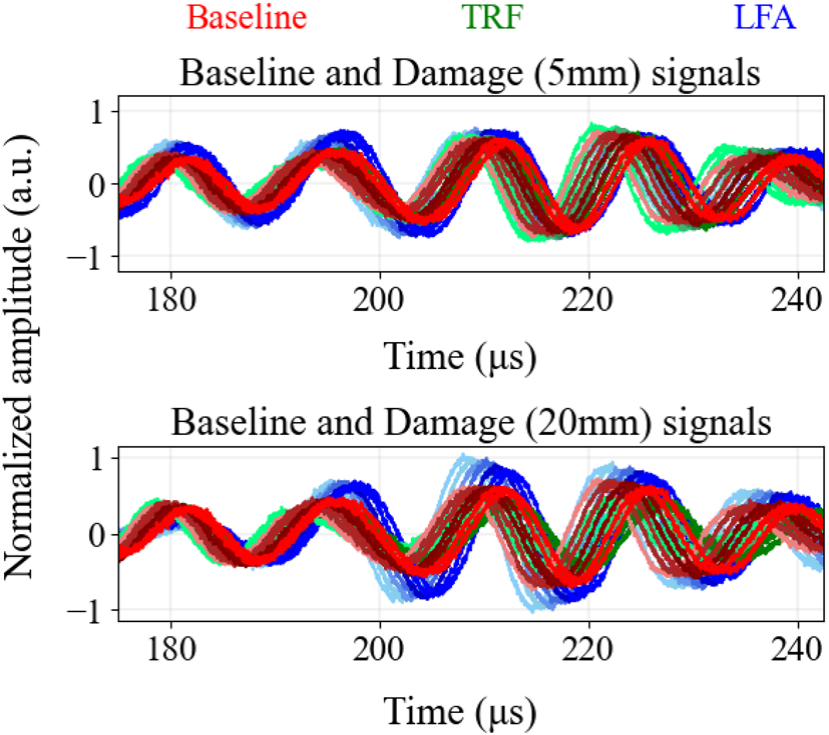
Noise-augmented data generated using FE simulations for temperature range 30–${90}\,^{\circ }\hbox {C}$, for baseline and TRF and LFA damage of 2 sizes: 5 mm and 20 mm. Lighter shades of colour represent data at lower temperatures and darker shades represent progressively higher temperatures. To better visualise the large extent of overlap across the various time-series, we show portion of the waveforms corresponding to A0 mode only.

Here, $\mathcal {[\varepsilon ]}$ is the piezoelectric permittivity matrix, [*C*] is the mechanical stiffness matrix, and [*e*] is the piezoelectric stress matrix. The density of the piezoelectric material was set to ${7500}\,{\hbox {kg}/\hbox {m}^{3}}$. The FE model contained 81 040 elements with 498 604 degrees of freedom. The simulation time step was chosen to be ${25}\,\hbox {ns}$ for $5-$cycle Hanning pulse actuation centered at ${75}\,\hbox {kHz}$, for total simulation time of ${400}\,\upmu \hbox {s}$. The computation time for each simulation on $64-$bit Intel Core-i$7-10$ 750 H 2.60 GHz CPU workstation with 16 GB RAM was ${4.15}\,\hbox {h}$. Simulations for TRF and LFA damages were conducted with the simulated damage with conditions similar to the experiment. The data were added with white and pink noise, in a manner similar to the noise-augmentation performed for experimental data to obtain SNR of 20 dB (Fig. 4).

### Feature extraction

In any damage detection approach, choosing the right features from GW signals is a crucial step since feature selection greatly affects accuracy of the damage assessment algorithm. Automated feature extraction using CNNs require training large networks that may not be feasible for edge implementation. While frequency-domain methods such as frequency decomposition and time-frequency methods such as spectrogram (obtained using short time Fourier transform) and scalogram (obtained using wavelet transform) are best suited to capture the non-stationary nature of GW signals^30^, they require substantial computation resources and are not feasible for embedded system implementation for edge-inference^31^. Features computed from time-domain signals acquired using the embedded system are therefore best suited for implementing ML models for damage inference at the edge. The utility of easy-to-compute statistical metrics obtained from time-domain signals for machine fault diagnosis was shown by Bandyopadhyay et al.^32^. For the damage assessment model presented in this work, we utilised 16 features computed from the time-domain signals, summarised in Table 2. Along with widely used statistical features such as mean absolute deviation (MAD), variance, standard deviation, root mean square energy (RMS), root mean square deviation (RMSD), kurtosis, skew, crest factor, impulse factor, shape factor and difference in peak to peak amplitude of signals, more advanced features based on signal energy^33^ were included to capture the impact of damage on amplitude and phase of the GW signals. MAD, variance, and standard deviation measure the deviation of the guided wave signal relative to the mean. However, these features only use one signal at a time, which does not allow comparison between two signals obtained for different states of the structure (e.g. healthy and damaged). Therefore, the remaining features were formulated by comparing a reference signal, usually derived from data for healthy structure taken at room temperature, to the signal obtained using the system in presence of damage or temperature variations.

**Figure 5:**
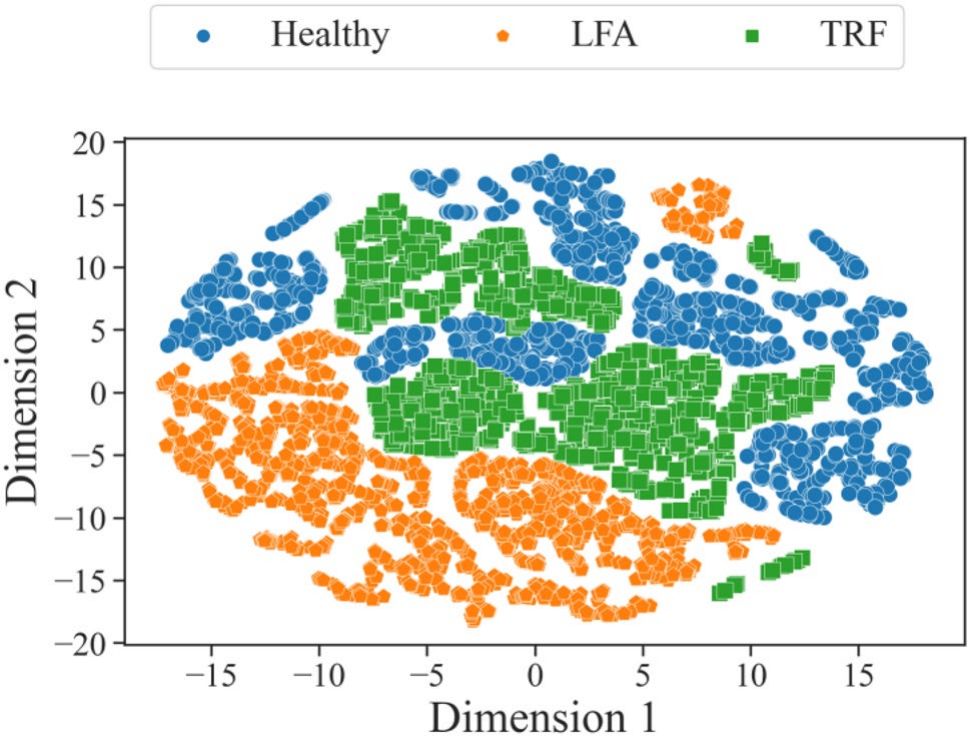
t-SNE plot of experimental data.

Variations in the experimental data were studied using T-distributed stochastic neighbour embedding (t-SNE) technique in two dimensions^34^. Constructing a t-SNE plot involves feature extraction followed by calculation of pairwise similarities among data points, which are then reduced to a lower-dimensional space using t-SNE. This reduction preserves relationships in the high-dimensional data, that can be easily visualised in two dimensions. In the resulting plot shown in Fig. 5, Dimension 1 and Dimension 2 represent transformed axes derived from the original high-dimensional space. These dimensions do not directly correspond to specific features, but capture nonlinear combinations preserving proximity relationships between data points. Thus, points located closer to each other in this two-dimensional visualisation possess greater similarity in the original higher-dimensional dataset. Interpreting clusters or structures in this reduced space offers insights into inherent relationships or groupings within the data, to visually identify underlying patterns. In this work, 16 specific features are already extracted from the time-series data, and the t-SNE plot is generated directly using these features. For generating the t-SNE plot in Fig. 5, features from vertical and horizontal paths of all three unit cells were used. Prior to t-SNE computation, the data were subjected to standard normalisation and feature scaling to ensure consistency across features. The t-SNE algorithm was configured with a perplexity value of 5 and was iterated 5000 times for convergence, utilising the Euclidean distance metric to measure data point relationships. The features of data obtained from damaged and undamaged paths are not linearly separable in this visualisation, and therefore an ML model is suitable for distinguishing damaged and healthy operating conditions.

**Table 2:** Features utilised for damage assessment.

Feature	Expression
Mean	$\frac{1}{n}\sum _{i=1}^{i=n}f(i)$
Median	$${\left\{ \begin{array}{ll} X[\frac{n+1}{2}], &{} n\text { is odd}\\ \frac{{X[\frac{n}{2}] + X[\frac{n}{2}+1]}}{2}, &{} n\text { is even} \end{array}\right. }$$
Mean absolute deviation	$\frac{1}{n}\sum _{i=1}^{i=n}|f(i) - \mu |$
Variance	$\frac{\sum _{i=1}^{i=n}{(f(i) - \mu )^2}}{n}$
Standard deviation	$\sqrt{\frac{\sum _{i=1}^{i=n}{(f(i) - \mu )^2}}{n}}$
Root mean square (RMS)	$\sqrt{(\frac{1}{n})\int ^n[f(i)]^2 dt}$
Root mean square deviation (RMSD)	$\sqrt{\frac{\int ^n[f(t)-f_b(t)]^2 dt}{\int ^n[f_b(t)]^2dt}}$
Kurtosis	$\frac{1}{n}\sum _{i=1}^{i=n}\left( \frac{x_i - \mu }{\sigma }\right) ^4$
Skew	$\frac{3(\mu - \mu _{1/2})}{\sigma }$
Crest factor	$\text {max}(|x_i|) \div \sqrt{\frac{\sum _{i=1}^{i=n} x_i^2}{n}}$
Impulse factor	$\text {max}(|x_i|) \div {\frac{\sum _{i=1}^{i=n} |x_i|}{n}}$
Shape factor	$\sqrt{\frac{\sum _{i=1}^{i=n} x_i^2}{n}} \div \frac{\sum _{i=1}^{i=n} |x_i|}{n}$
Peak to peak	$A-A_b$
Ratio of signal energy	${\frac{\int ^T{|f(t)|^2}dt}{\int ^T{|f_{b}(t)|^2}dt}}$
Damage Index	${\frac{\int ^T{|f(t)-f_{b}(t)|^2}dt}{\int ^T{|f_{b}(t)|^2}dt}}$
Normalised difference of signal energy	${\frac{\int ^n{|f(t)|^2}dt-\int ^n{|f_{b}(t)|^2}dt}{\int ^n{|f_{b}(t)|^2}dt}}$

Glossary: *f*(*i*): data samples in the signal, $i=1,2,\dotsc ,n$, *n*: length of time series, *X*: ordered list of values in the series, $\mu$:mean value of samples in time-series *f*,$\sigma$: standard deviation of samples in time-series *f*, $f_b$: baseline signal, *A*: peak to peak amplitude of signal *f*, $A_b$: peak to peak amplitude of baseline signal $f_b$.

### Unsupervised learning

Generating extensive labeled data to capture all possible damage scenarios under all possible varying environmental and operating conditions is impractical, and thus significantly limits the applications of supervised learning algorithms for GW-SHM. Therefore, we have explored the use of unsupervised learning methods to overcome these limitations. One such approach is anomaly detection, which involves finding outliers in data that were not represented in the training data, and mark them as possible damage scenarios. This involves a neural network that is trained for an approximate reproduction or reconstruction of the input signal by passing it through a series of neural layers. This method is different from supervised learning, because there are no separate discrete (classification) or continuous (regression) labels associated with input data. Training an unsupervised learning model using only data obtained for baseline operation (healthy or undamaged condition) at various temperatures would enable the model to learn variations due to temperature. This would in turn be used to distinguish the same from variations due to damages, which would be identified as outliers, since data from damaged conditions would not be part of the training exercise. Anomalies in the data due to damage will result in higher error in reconstruction, and therefore be identified as data belonging to damaged condition. The reconstruction error is quantified by the mean square error (MSE) between reconstructed and input data:

1 $$\begin{aligned} MSE = {\frac{1}{n}} \times {\sum _{j=1}^{j=n}{\left( a_{j} - \bar{a_{j}}\right) ^2}} \end{aligned}$$

where *n* denotes number of elements in the input data, ${a_{j}}$ is the $j^{th}$ element in the input data and ${\bar{a_{j}}}$ is $j^{th}$ element in the reconstructed output data. The accuracy of the model is calculated by setting the reconstruction error threshold for determining anomaly as $\mu + \sigma$, where $\mu$ and $\sigma$ are the mean and standard deviation respectively of the distribution of reconstruction error obtained from training with healthy data.

Our study focuses on assessing damage using features extracted from data obtained from damaged and undamaged regions. We employ an unsupervised learning model trained solely on the baseline data features. This choice of using hand-crafted features instead of raw time-series data helps in reduction of the model size, by enabling us to forego convolutional layers, and exclusively construct the model using densely connected layers. All data were normalised such that all values lie in the range of $[-1,1]$, and the normalised data were used for noise augmentation and feature extraction. Since we calculated 16 features from each time-series, the input dimension of the model is 16. The first layer in the model comprises a dense configuration with 16 input and 16 output nodes. Subsequently, we expand the output nodes to 32, followed by 64 nodes. Next, we incorporate a dropout layer to avoid over-fitting. To reconstruct the input features, we mirror the preceding layers, employing a dense layer with 64 output nodes, followed by one with 32 and then a layer with 16 output nodes. Consequently, the network yields 16 reconstructed features. The model architecture is summarised in Table 3. The model was developed in Python using TensorFlow library and Keras environment. The network was trained using rectified linear unit (ReLU) activation function and Adam optimizer. Keeping the number of neurons fixed, the batch size, learning rate and number of epochs were optimised using *RandomSearch* algorithm executed for 10 iterations. The best set of parameters was then selected based on maximum accuracy obtained with $5-$fold cross-validation score. The range of hyper-parameter values passed to the algorithm and optimal values thus obtained are listed in Table 4.

## Results and discussion

Instead of setting up a separate model for learning features from vertical and horizontal paths, a single model was trained on combined data from horizontal and vertical paths, to reduce the computational burden on the embedded edge platform. The performance of the model was evaluated for experimental and simulation datasets. To calculate the 16 features from time-series data, segments corresponding to the initial ${200}\,\upmu \hbox {s}$ portion of the time-series were utilised. Note that the A0 mode is expected to be most sensitive to damage. However, we have also included portions corresponding to other GW modes, so that a generic model may be developed, that would not require prior knowledge of material properties of the structure under test, for determination of group velocity and thereby arrival time of a desired GW mode.

**Table 3:** Summary of model architecture.

Layer (# filters)	# Parameters
Dense (16)	272
Dense (32)	544
Dense (64)	2112
Dropout	0
Dense (64)	4160
Dense (32)	2080
Dense (16)	528
Total trainable parameters	9696

**Table 4:** Hyperparameter tuning using RandomSearch.

Hyperparameter	Range	Optimal value
Learning rate	[0.001, 0.01, 0.1]	0.01
Batch size	[16, 28, 32, 64]	32
No. of epochs	[50, 100, 150, 200]	150

### Analysis of experimental data

**Figure 6:**
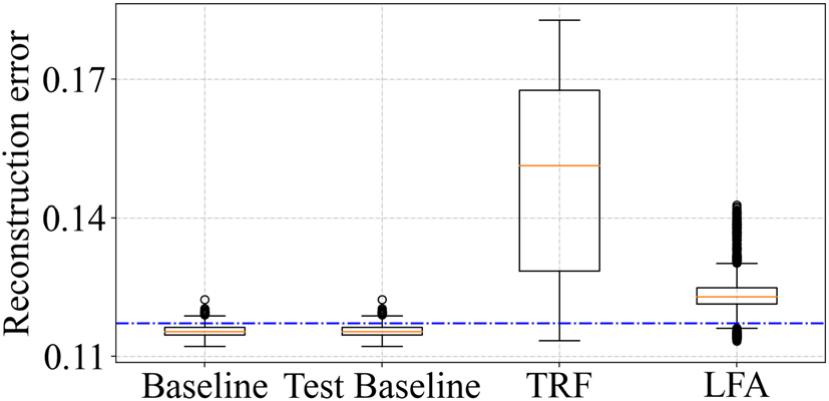
Reconstruction error for experimentally generated data.

Experimental data collected on undamaged panel were used for training the model, which was then tested on data collected on panel with TRF and LFA damage. A total of $50 \times 19 \times 6 = 5700$ time-series recordings were available for each of baseline, TRF and LFA conditions: 50 noise augmented copies generated at 19 temperatures varying from 0 to ${90}\,^{\circ }\hbox {C}$ for 6 paths. Data samples corresponding to undamaged (i.e., baseline) condition were randomly split into 50% for training, 20% for validation, and the remaining 30% for testing the model. Training the model required ${5.58}\,\hbox {s}$ on Intel Core-i$5-10$ 500 CPU workstation with 16 GB RAM. We verified that the training loss as well as validation loss decrease smoothly with increasing number of epochs, confirming that the model is not over-fitting to the data. After training, we tested the model performance on another independently collected dataset consisting of data from undamaged, as well as damaged portions. Figure 6 shows distribution of reconstruction error for model predictions for these experimentally generated data for test data of the baseline set used for training, and the independently recorded dataset for testing the model, comprising of baseline, TRF and LFA conditions. The paths that do not contain damage in-line (i.e., *baseline* and *test baseline*) are correctly identified, as the reconstruction error is lesser than the $\mu + \sigma$ threshold. Similarly, damaged paths TRF and LFA are also identified correctly, as the reconstruction error is greater than the $\mu + \sigma$ threshold. Accuracy and F1 score for these results are shown in Table 5. These results suggest that the chosen set of input features are suitable for capturing the variation in data due to temperature, while simultaneously enabling the model to distinguish changes due to presence of damage.

**Table 5:** Accuracy and F1 score for experimental data.

Case	Accuracy (%)	F1 score (%)
Test Baseline	99	99
TRF	95	86
LFA	95	83

**Figure 7:**
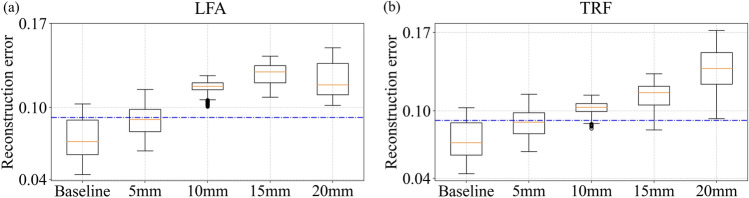
(**a**) Reconstruction error for LFA and (**b**) reconstruction error for TRF data generated by FE simulations.

### Analysis of simulation data

To study the performance of the damage identification for temperature-affected and noise-augmented data corresponding to various damage sizes, FE simulation data were generated using COMSOL Multiphysics (with noise augmentation performed using MATLAB). A total of $50 \times 7 \times 3 =1050$ datasets were available for training and validation of the model: 50 noise augmented copies were generated for each of the 7 temperatures varying from 30 to ${90}\,^{\circ }\hbox {C}$ for 3 paths. The same model architecture as used for evaluating experimental data was used for analysing these simulation generated data, with the only exception being the batch size, which was set as 28, since we observed the model to over-fit to the data for batch size of 32. Figure 7 shows distributions of reconstruction error for model predictions for LFA and TRF defects of various sizes. The corresponding accuracy and F1 score metrics are reported in Table 6. Since defects of larger sizes produce larger changes in signal amplitude, the model is able to easily discern TRF and LFA damages of sizes 10 mm and larger. The increasing trend of reconstruction error with defect size indicates that the absolute value of reconstruction error could possibly be used as an indicator of defect size (i.e., damage intensity). Defects of size 5 mm are not as accurately classified as damaged. Note that the sensitivity of the model to smaller damages could potentially be improved by exploring transduction at higher frequencies. However, the PZT transducers used in this study were found to have degradation in transduction efficiency at frequencies higher than 150 kHz, and therefore only 75 kHz actuation was explored in this work.

**Table 6:** Accuracy and F1 score for simulation generated data.

Damage size (mm)	TRF		LFA	
	Accuracy	F1 score	Accuracy	F1 score
5	50	35	50	35
10	90	73	96	88
15	90	73	96	88
20	96	88	96	88

### Edge implementation of trained model

**Figure 8:**
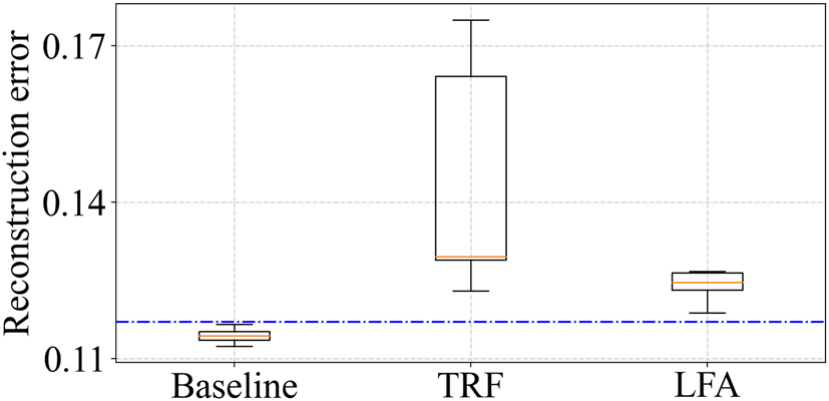
Reconstruction error for experimental data for edge implementation of model.

The trained model was deployed using TensorFlow Lite framework on the MicroBlaze$^{\circledR }$ RISC processor core in the Xilinx Artix$^{\circledR }$-7 device (Digilent Cmod A7-35T module). The challenge in implementing machine learning model in such a device is the absence of a processor core which is required to perform complex control tasks and run TFLite. The MicroBlaze CPU is a family of highly configurable, drop-in, modifiable preset $32-$bit Harvard RISC microprocessor architecture optimised for implementation in Xilinx FPGAs. MicroBlaze is fully supported by Eclipse based integrated development environments (IDEs) Xilinx SDK/Vitis, which allow direct creation of an application project for the Cmod A7-15T module and auto-generation of all board support package (BSP) files. The corresponding TFLite kernels were also modified to invoke the custom IPs for deploying the trained model on MicroBlaze. The hardware for FPGA is configured and bitstrean is generated using Vivado 2019.1. Once the bitstream is generated, BSP is created to provide all low level drivers for the hardware using SDK 2019.1. The TensorFlow Lite model header file generated using Python application programming interface (API) *TensorFlow Lite converter* is imported in the SDK project, and stored as C byte array in read-only program memory on the FPGA device. For more details on the implementation, please see source code available at^35^, which describes the use of this framework for handwritten digit classification in MNIST database and^36^, which describes the use of this framework for implementing a reinforcement learning model. The model architecture described in Table 3 was deployed on the device and the performance was evaluated on the same test cases used for evaluating model performance on experimental data. We observed similar accuracy as that reported in Table 5, with average inference time approximately ${900}\,\upmu \hbox {s}$ for 100 MHz clock frequency, and measured power consumption of 0.336 W. The resource utilisation in the FPGA is summarised in Table 7, and a plot of reconstruction error for edge implementation is shown in Fig. 8.

**Table 7:** Resource utilisation for model implementation on FPGA device for experimental data.

Resource	Utilised	Available	Utilisation (%)
LUT	5463	20 800	26.26
FF	4352	41 600	10.46
BRAM	49	50	98
DSP	5	90	5.56

**Table 8:** Comparison of this work with other unsupervised learning algorithms for GW-SHM reported in literature.

References	Application	Architecture	Model size and edge-compatibility
Lee et al.^17^	Fatigue damage detection and classification in composite structures	1D deep AE	Model size not reported; model not edge-compatible
Sawant et al.^18^	Damage identification and localisation in OGW dataset	1D CAE with transfer learning	1.1M parameters; model not edge-compatible
Eybpoosh et al.^37^	Damage detection in pipelines under varying environmental and operational conditions	K-means, SVM and KL divergence	Model sizes not reported; models not edge-compatible
Zhang et al.^38^	Automated damage severity and orientation detection	SVM	Model size not mentioned; accuracy drop on prediction of noise augmented data; model not deployed on edge device
Rai et al.^39^	GW-based semi-supervised damage diagnosis in composite laminates using ResNet autoencoder with transfer learning	1D CAE	$\approx 260$k trainable parameters with transfer learning (Note: ResNet CAE is not edge-compatible)
Rautela et al.^40^	Delamination detection in composite panels	2D CAE	$\approx$ 1.2 M trainable parameters (encoder) and $\approx$ 1 M trainable parameters (decoder)
Yang et al.^41^	Assessment of damage under regular and irregular variations of EOCs in aluminum plate	AE	Model not edge-compatible
Sikdar et al.^42^	Assessment of breathing-like debonds in lightweight stiffened composite panels	Deep CNN	Model not edge-compatible
**This work**	TinyML enabled temperature-compensated damage detection on edge device in HCSS	ANN	9696 parameters; model deployed on Cmod A7-15T FPGA module

### Discussion

Limited memory and computational resources available on embedded edge devices make it challenging to design and deploy machine learning tools for GW-SHM, as arbitrarily large models with significant amount of signal pre-processing typically presented in literature are rendered impractical. This is especially true for permanently deployed SHM systems, as compared to solutions for traditional periodic inspection and nondestructive testing and evaluation (NDT &E). The application of TinyML for GW-SHM presented in this work illustrates the potential for further exploration towards implementation of autonomous damage inspection strategy on resource constrained hardware. No knowledge of material properties of the structure or impact of damage on guided wave propagation is necessary to apply this method, and therefore it is also potentially scalable to various types of damages in other structures. However, this would require careful examination of damage-sensitive features that must be calculated from the time-series data and provided as input to the model. Generating the 16 hand-crafted features from experimentally acquired data reported in this work, and computing reconstruction error takes only a few milliseconds. While the performance can be optimised further using a more capable albeit expensive FPGA device, the Cmod A7-15T module offers an excellent trade-off between performance and price (<USD $\$ 100$). A comparison of our method to other unsupervised learning based damage assessment methods in GW-SHM reported in literature is presented in Table 8. Compared to many of these implementations, our model is extremely light-weight (<10 000 trainable parameters) and can therefore be effectively stored and executed on the edge device used for configuration and transduction of GW signals. The model inference time on the edge device is <1 ms, thereby enabling efficient duty-cycling of such an SHM solution, helping conserve power and battery life in the field. Choosing suitable features allows such a light-weight model to distinguish changes in data due to damage from those due to temperature variation. Thus, our work presents an important breakthrough of relevance to the GW-SHM community for realising practically relevant smart-sensor nodes that may be permanently deployed on infrastructure.

## Conclusion

In summary, we present a lightweight unsupervised learning algorithm framework that is deployed on an FPGA device for end-to-end implementation of a GW-SHM embedded system for data acquisition, storage, feature extraction and damage assessment. The variance in peak signal amplitude, variation in signal energy, and variation in signal from baseline are all captured by the 16 hand-crafted features used to represent the time-domain data. These features are adaptable to a wide range of GW-SHM applications because they capture the typical impact of damages on guided wave propagation in structures. The method was tested using data generated from FE simulations on HCSS panels equipped with PZT-5H transducers and an experimental setup for inducing temperature change in the range of 0–${90}\,^{\circ }\hbox {C}$. The unsupervised machine learning model used for damage assessment consists of only 9696 parameters, and shows high accuracy when trained and tested with noise augmented data with 20 dB SNR. The trained model was also successfully deployed on the MicroBlaze RISC processor core in the Xilinx Artix-7 FPGA device. Despite the limited number of features and small model size employed for edge implementation, the models evaluated in this work showed reasonably high accuracy for disbond and delamination defects. We aim to investigate feasibility of the method proposed in this work for different types of structures, damages and severity. In future work, we shall also explore strategies to improve the sensitivity of this framework to defects of smaller sizes and incorporate online training on the edge device to realize completely autonomous self-adaptable GW-SHM systems.

## Data Availability

The datasets used and analysed during the current study available from the corresponding author on reasonable request.
